# Bootstrap Resampling of Temporal Dominance of Sensations Curves to Compute Uncertainties

**DOI:** 10.3390/foods10102472

**Published:** 2021-10-15

**Authors:** Shogo Okamoto

**Affiliations:** Department of Computer Sciences, Tokyo Metropolitan Universities, Hino, Tokyo 191-0065, Japan; okamotos@tmu.ac.jp

**Keywords:** temporal dominance of sensations, confidence interval, standard error, Markov chain

## Abstract

In the last decade, temporal dominance of sensations (TDS) methods have proven to be potent approaches in the field of food sciences. Accordingly, thus far, methods for analyzing TDS curves, which are the major outputs of TDS methods, have been developed. This study proposes a method of bootstrap resampling for TDS tasks. The proposed method enables the production of random TDS curves to estimate the uncertainties, that is, the 95% confidence interval and standard error of the curves. Based on Monte Carlo simulation studies, the estimated uncertainties are considered valid and match those estimated by approximated normal distributions with the number of independent TDS tasks or samples being 50–100 or greater. The proposed resampling method enables researchers to apply statistical analyses and machine-learning approaches that require a large sample size of TDS curves.

## 1. Introduction

Over the last decade, temporal dominance of sensations (TDS) methods, in which multiple types of temporally evolving subjective responses are recorded, have been proven to be effective methods of sensory appraisal in the field of food sciences by many researchers [1,2,3]. Furthermore, in some recent studies, TDS methods have been applied to tasks involving visual, auditory, and haptic cues [4,5,6,7]. TDS methods are becoming a standard of sensory appraisal, irrespective of the type of modality.

Typically, in the task of the TDS method, adjective descriptors (Except for adjective descriptors, onomatopoeic words are used, for example [8]) listed on a computer screen are sequentially selected by assessors. These descriptors correspond to the dominant sensations felt while experiencing food stimuli. The dominant sensation is defined as “the sensation that catches his/her attention” [9] or constitutes the intensity of or change in the sensory profile [10]. In most data analysis methods, the period that each descriptor is selected as dominant is calculated; in other words, this is the dominance duration [8,11,12,13]. These periods are treated as variables, and principal component analysis or canonical variate analysis are then applied to evaluate the differences among the food stimuli. Furthermore, as the most typical analysis [9], by accumulating the results of independent TDS tasks, a proportion that a certain descriptor is dominant in is computed at each instant. These proportions are functions of continuous time and are displayed as “TDS curves.” These TDS curves are visually inspected, and their key values, such as maximum values and the time when the maximum values are observed, are calculated and compared using multivariate analysis techniques among different food products [2,14,15].

In the standard data analysis method established by earlier studies, a set of TDS curves are computed using the results of all the independent tasks. This method of curve production prevents the use of many analytical methods requiring a large sample size. For instance, if we are interested in the peak curve value of a certain descriptor, there is no standard method to estimate how it fluctuates, meaning we cannot judge whether the peak value is different from a hypothesized value. Further, in general, machine-learning techniques to predict or utilize TDS curves require a large set of TDS curves. To acquire multiple sets of TDS curves, for example, TDS tasks need to be repeated by the same panels for the same food stimuli [15]. These multiple sets of TDS curves are necessary to consider the curves’ uncertainties for some statistical analyses, including hypothesis testing. Such repetitions are acceptable for acquiring a few sets of TDS curves; however, more curve sets are required for some analysis techniques. To circumvent this problem, bootstrap resampling methods can be used [16,17,18]. In these resampling methods, as described in Section 2.2, tasks are sampled with replacement from a set of independent TDS tasks, and many sets of TDS curves are acquired. For instance, in [17], to apply the principal motion analysis on TDS curves, dozens of TDS curve samples were produced by bootstrap resampling to avoid overfitting. However, thus far, the validity of bootstrap resampling of TDS curves has remained unclear.

Bootstrap resampling for TDS curves yields some benefits. First, it allows us to adopt analytical methods that require many curve samples. Second, bootstrap resampling enables the estimation of curve uncertainties. More specifically, we can test whether a curve value at a certain instance is significantly greater than a hypothesized value. Further, a hypothetical test of curve values between two or multiple different descriptors can be conducted. Nonetheless, Pineau et al. [2] suggested that the uncertainties of TDS curves can be estimated based on approximated normal distributions because TDS curve values correspond to proportions. Provided that the uncertainties estimated by bootstrap resampling and normal distributions match, the validity of bootstrap resampling is supported.

However, the validity of the bootstrap resampling method for TDS curves has yet to be studied. As such, the present study proposes a bootstrap resampling method for estimating the uncertainties of TDS curves, demonstrates the method’s validity, and calculates the necessary sample size. In particular, our interest is how many samples are necessary to accurately estimate the uncertainties of TDS curves and to create random TDS curves, of which fluctuations represent the uncertainty of the population. As the most popular indices of uncertainties, 95% confidence intervals and standard errors are computed using the distribution of TDS curve values that are generated by resampled data. These uncertainties are validated via comparison with the population produced via Monte Carlo simulation, where Markov chain models that simulate TDS tasks [19,20,21] are used to produce a large number of random samples. The present study is the first attempt to utilize Markov chain models and Monte Carlo simulation to yield a large dataset of TDS curves.

## 2. Methods

### 2.1. TDS Method

Herein, the typical procedures of TDS tasks and computation of TDS curves are introduced to help understand the remaining part of the paper. Regarding further details, earlier publications [1,2,3,9] are referred to.

**Figure 1:**
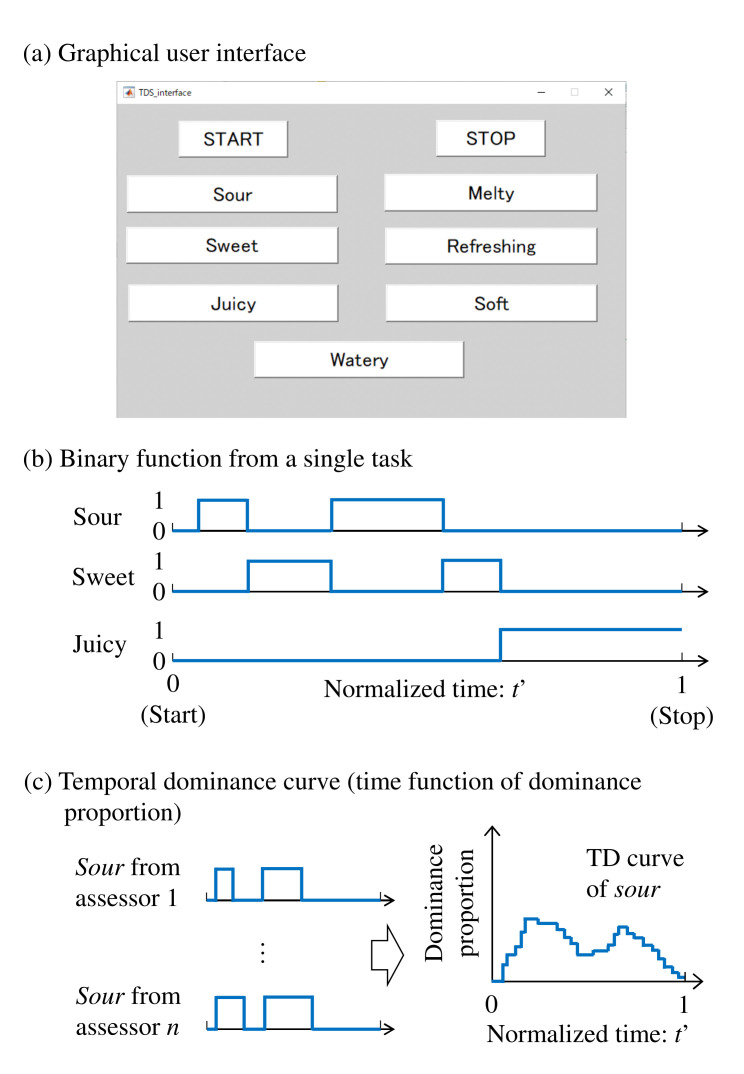
Temporal dominance task. (**a**) Graphical user interface. (**b**) Binary function of selected/non-selected from a single TDS task. (**c**) Temporal dominance curves as the accumulation of binary functions by descriptors. Partly adapted from [18].

In TDS tasks, a graphical user interface is used as shown in Figure 1a. The assessor pushes the start button at the moment of putting the food in his/her mouth. S/he then pushes the button corresponding to the sensation that comes to mind. Different buttons are sequentially pushed as the dominant sensations change. Once the button is pushed, it remains selected until the next button is pushed. The stop button is pushed when the food vanishes in the mouth or prominent sensations fade after swallowing the food. These procedures comprise a single TDS task. As shown in Figure 1b, from a single task, that is, the *i*-th task, binary functions of continuous time that take either 0 (unselected) or 1 (selected) are yielded for individual *p* descriptors:

(1) $$\begin{array}{c} b_{ij}\left(t\right)\in \left\{0,1\right\}\;\left(j=1,...,p\right), \end{array}$$

where *j* is the index of descriptors.

This task is repeated by *n* assessors. TDS tasks do not necessarily require well-trained or professional assessors [3,15,22]. Typically, only familiarization with the TDS tasks and comprehension of the descriptors used in the tasks are required [9,23]. Based on the results of *n* tasks, the proportion at which each descriptor is dominant at moment *t* is computed. For this computation, typically, the period of each task is normalized such that the time elapsed between pushing the start and stop buttons is equal to 1. The normalized binary functions are accumulated for individual descriptors. As shown in Figure 1c, the dominance proportions are computed by dividing the accumulated values by *n*.

(2) $$\begin{array}{c} d_{j}\left(t^{\prime }\right)=\sum_{i=1}^{n}\frac{b_{ij}\left(t^{\prime }\right)}{n}, \end{array}$$

where $t^{\prime }$ is the normalized time ( $t^{\prime }\in \left[0,1\right]$ ). The time function $d_{j}\left(t^{\prime }\right)$ is the TDS curve for descriptor *j*.

### 2.2. Bootstrap Resampling of TDS Tasks

Herein, the bootstrap resampling method for TDS tasks is introduced, which follows a standard bootstrap resampling method [24]. Note that this method is very different from bootstrap resampling methods for time-series data, such as block bootstrap [25,26]. It is unique to the temporal dominance tasks and has been adopted in some earlier studies [16,17,18].

The results of *m* independent temporal dominance tasks for the same food are collected. We call this sample set $P_{0}$ . The set of TDS curves computed using all the samples in $P_{0}$ is called $C_{0}$ . From $P_{0}$ , *m* tasks are randomly sampled with replacement and form a new sample set $P_{1}$ . Note that some tasks in $P_{1}$ may be the same. A set of TDS curves, $C_{1}$ , is computed based on $P_{1}$ . This process of resampling is repeated *q* times, and *q* curve sets $C_{1},...,C_{q}$ are acquired. The expected values of *q* curve sets match the values of curves in $C_{0}$ .

### 2.3. Monte Carlo Simulation of Temporal Dominance Tasks Based on Markov Chains

The combination of Monte Carlo simulation and Markov chains is used to simulate a large number of TDS tasks. The Monte Carlo method is a simulation based on many random samples, while Markov chains express the probabilistic transitions of dominant sensations while eating food [19,20,21,27] and are used to simulate TDS tasks.

**Figure 2:**
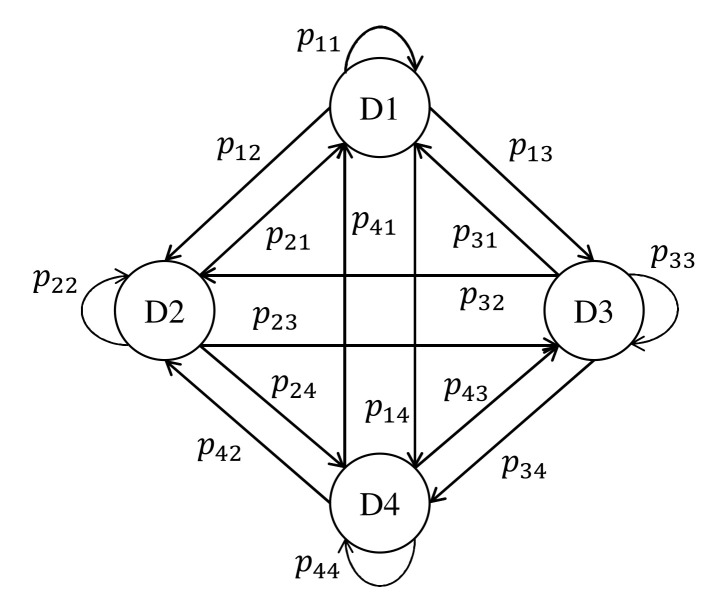
Markov chain model for Case 1 involving four descriptors. D1–D4 indicate descriptors 1–4. $p_{ab}$ indicates the probability of transitioning to D*b* from D*a*.

In the present study, two cases are simulated. In Cases 1 and 2, four and six descriptors are used, respectively. As shown in Figure 2, descriptors are expressed as states, and the initial state at $t^{\prime }=0$ is determined by the initial distribution of (D1, D2, D3, D4) = (0.5, 0.25, 0.25, 0) for Case 1 and (D1, D2, D3, D4, D5, D6) = (1/6, 1/3, 1/3, 1/6, 0, 0) for Case 2. Here, D*i* ( i∈{1,2,3,4} for Case 1 and i∈{1,2,3,4,5,6} for Case 2) indicates descriptor *i*. For example, in Case 1, D1 is selected at the probability of 0.5 at $t^{\prime }=0$ . The state at $t^{\prime }=\Delta t$ after one transition is probabilistically determined by the transition probabilities in Table 1 and Table 2. For simplification, each task is assumed to consist of 20 transitions; that is, Δt=1/20 . The state-transition table varies according to $t^{\prime }$ . The entire period is split into three phases: initial ( $t^{\prime }=0$ –6/20), middle ( $t^{\prime }=7/20$ –13/20), and last ( $t^{\prime }=14/20$ –1.0). The transition probabilities in the tables were arbitrarily determined by the author. For example, when D1 is selected at $t^{\prime }=0$ , D1 is likely to be kept selected at $t^{\prime }=\Delta t$ at the probability of 0.5, according to Table 1. D2 is also likely to be selected after D1 at the probability of 0.3. By continuing this process along the timeline, the temporal evolution of descriptors is acquired. Consequently, the process to sequentially select descriptors referring to dominant sensations is simulated.

**Figure 3:**
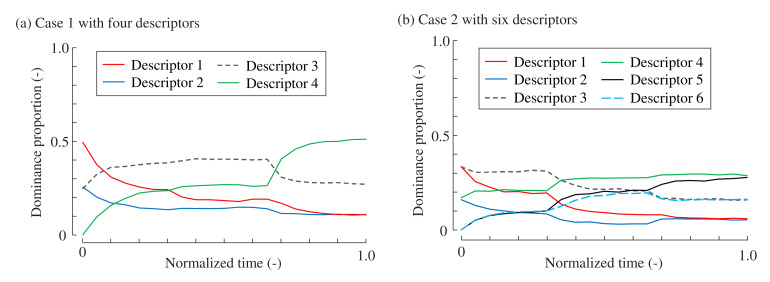
Temporal dominance curves, that is, dominance proportions, computed from the populations of which *n* = 10,000. (**a**) Case 1 with four descriptors. (**b**) Case 2 with six descriptors.

Following the abovementioned methods, for both Cases 1 and 2, the results of *n* tasks are produced and treated as the population. Figure 3 shows the TDS curves computed by all the tasks in the population when n=10000 .

### 2.4. Estimation of 95 % Confidence Intervals and Standard Errors

As mentioned above, as representative indices of uncertainties, 95% confidence intervals and standard errors are adopted. First, these values are estimated using resampled TDS curves. Second, these values are estimated using approximated normal distributions. The values estimated by the two approaches are then compared.

**Figure 4:**
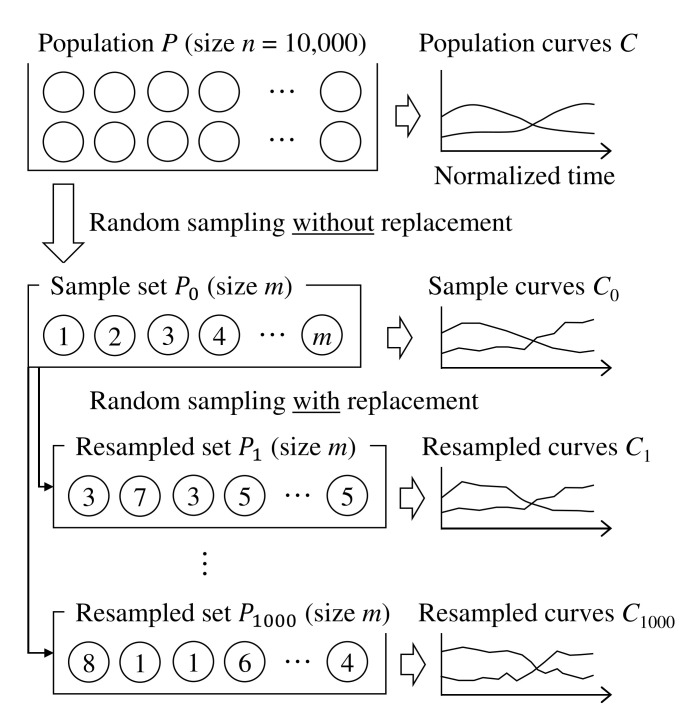
Method of simulation. Individual circles indicate independent TDS tasks.

As in Figure 4, *m* tasks are randomly sampled from the population, including 10,000 tasks: *n* = 10,000. Hence, *m* is the sample size and is 15, 30, 50, 75, 100, 150, 200, 250, 300, 350, 400, 450, or 500. Based on *m* samples, the bootstrap resampling procedure is repeated 1000 times ( q=1000 ), and 1000 sets of TDS curves are computed ( $C_{1}$ , …, $C_{1000}$ ). At each instant $t^{\prime }$ , for each descriptor, the 95% confidence interval is estimated as the range between the 25th largest curve value and 25th smallest curve value among the 1000 curve values. Then, it is checked whether the dominance proportion value in the population is included in this range. These procedures are repeated 1000 times, and the proportion for which the population value was included in the estimated range is calculated. Note that this proportion is expected to be close to 0.95.

Similar processes are conducted for standard errors. The range between the 159th largest and 159th smallest values is used as the estimated standard error. The proportion at which the population value is included in the estimated range of standard error is expected to be close to 0.682.

The values of TDS curves are thought to be subject to normal distributions, because the values are proportions [2]. The uncertainties can then be computed using normal distributions. When the curve value for descriptor *i* at time $t^{\prime }$ is denoted by $d_{i}\left(t^{\prime }\right)$ , the 95% confidence interval of $d_{i}\left(t^{\prime }\right)$ is estimated by

(3) $$\begin{array}{c} d_{i}\left(t^{\prime }\right)\pm 1.96\sqrt{\frac{d_{i}\left(t^{\prime }\right)\left(1-d_{i}\left(t^{\prime }\right)\right)}{m}}. \end{array}$$

Similarly, the standard error is estimated by

(4) $$\begin{array}{c} d_{i}\left(t^{\prime }\right)\pm \sqrt{\frac{d_{i}\left(t^{\prime }\right)\left(1-d_{i}\left(t^{\prime }\right)\right)}{m}}. \end{array}$$

## 3. Results

**Figure 5:**
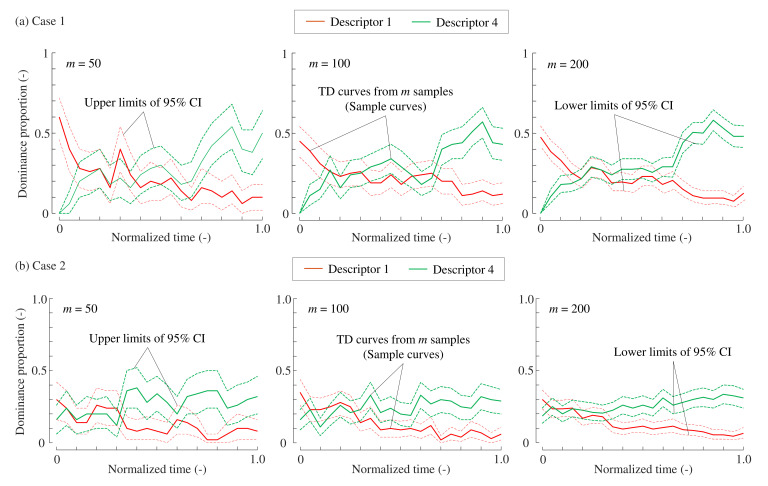
Examples of temporal dominance curves and 95% confidence intervals for Cases 1 (**a**) and 2 (**b**). For visual clarity, only Descriptors 1 and 4 are shown.

Figure 5 shows the examples of sample TDS curves and confidence intervals that were estimated by resampling. The three figures show those simulated when the sample sizes are m=50 , 100, and 200, respectively. Following the principles of statistical estimation, a greater sample size leads to smaller confidence intervals. Although they are not shown, this trend is the same for the standard errors.

**Figure 6:**
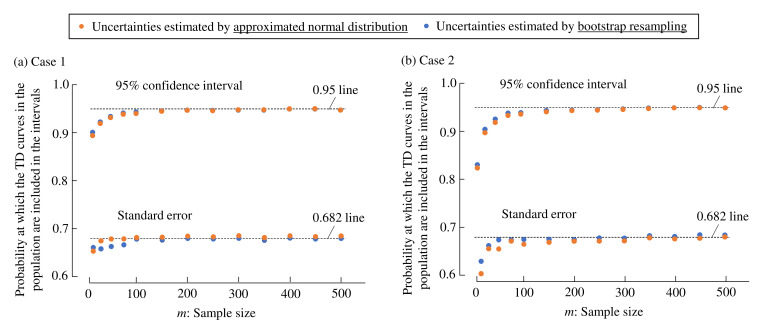
Probabilities that the dominance proportions in the population are included in the estimated uncertainties, that is, confidence intervals and standard errors. (**a**) Case 1. (**b**) Case 2. The uncertainties are computed using resampling and normal distributions. Probabilities are computed at m= 15, 30, 50, 75, 100, 150, 200, 250, 300, 350, 400, 450, and 500.

Figure 6 shows the inclusion probabilities that the dominance proportions of the population are included in the estimated confidence intervals and standard errors at each discrete moment $t^{\prime }$ for different *m* values. Note that the uncertainties were computed by the two methods. One was based on resampling, and the other was based on normal distributions following (3) and (4). When *m* was relatively small, the inclusion probabilities were smaller than the expected values, that is, 0.95 and 0.682 for confidence intervals and standard errors, respectively. The confidence intervals and standard errors were underestimated with small *m* values. Note that it is empirically known that uncertainties based on approximated normal distributions are inaccurate when mp<5 or m(1−p)<5 , where *p* is the sample proportion, suggesting that estimated uncertainties are not reliable for small sample sizes. As in the figures, when *m* was large, the uncertainties estimated by resampling and normal distributions were close to each other.

Comparing Case 1 (four descriptors) and Case 2 (six descriptors), the inclusion probabilities of Case 1 seem larger than those of Case 2 when m=15 and 30. The present study does not further investigate this point. Note that the differences between the two cases include the number of descriptors and profiles of TDS curves. Hence, the root causes of the differences in the inclusion probabilities between the two cases remain unclear.

## 4. Discussion

Two types of uncertainties, standard error and 95% confidence interval, of TDS curve values were repeatedly estimated through a combination of Monte Carlo simulation and bootstrap resampling. Approximately 68.2% of the estimated standard errors included the population values. Similarly, 95% of the estimated confidence intervals included the population values. These results indicate that resampling of TDS tasks is valid for estimating uncertainties with large sample sizes *m*. Thus, the resampling produces random TDS curves centering around the sample curves. In terms of the acceptable *m* values, based on Figure 6, when m≥100 , the uncertainties were fairly estimated. However, practically, the sample size of 100 is unlikely for most settings of sensory appraisal. Most of the previous studies on temporal dominance methods do not satisfy this sample size. Considering this situation, the present study proposes an acceptable value range of *m* = 50–100 while noting that the uncertainty indices tend to be underestimated with small *m* values.

As mentioned above, bootstrap resampling is beneficial for analysis techniques requiring many TDS curves. Because of the bootstrap method, temporal dominance methods and a variety of statistical and machine-learning techniques will be closely tied. Typically, the concern of overfitting can be circumvented by using resampled curves. Another suggestion of the present study is that hypothesis testing of dominance proportions or values of TDS curves can be conducted via approximated normal distributions. A standard parametric method for proportions is available when the sample size is large. For example, a dominance proportion at $t^{\prime }$ for a certain brand of food can be statistically compared with that for another brand. Further, a dominance proportion at $t^{\prime }$ is tested with regard to whether it is significantly greater than zero or the chance level. Note that these hypothesis tests using approximated normal distributions should involve a sample size above 50–100, although such restrictions on the sample size were not mentioned in the original study by Pineau et al. [2] nor in the international standards [9].

Some limitations of this study should be mentioned. First, the acceptable *m* values may depend on populations, but only two types of population models were investigated in this study. Particularly, when the TDS curves of the population include small values of nearly zero, it is difficult to accurately estimate uncertainties. However, such small values below the chance level are typically regarded as trivial and ignored for analyses [2]. Second, this study is limited to the TDS method, where only one descriptor can be selected at each moment, and the dominance proportions can be computed. As a variation of the TDS method, the temporal check-all-that-apply (TCATA) method [28,29] is also popular, as multiple descriptors can be selected simultaneously. Although TDS and TCATA methods [30,31] are often compared, the curves yielded by the TCATA method do not indicate dominance proportions. Moreover, thus far, mathematical models that can be used for a Monte Carlo simulation of the TCATA method have not been determined, whereas Markov chains are used for TDS methods [19,20,21].

## 5. Conclusions

The TDS method is a time-series sensory appraisal method that has been increasingly used in the field of food science. In general, a set of dominance-proportion curves is computed based on all the samples, whereas some statistical and machine-learning techniques require several or many sets of curves. Furthermore, it is thought that the uncertainties of dominance proportions are computed using approximated normal distributions; however, their validity is yet to be investigated. Hence, thus far, the estimation of uncertainties has rarely been utilized. Resampling is expected to resolve these issues, and this study proposes a resampling method for TDS curves. Through Monte Carlo simulation, the 95% confidence intervals and standard errors could be adequately estimated when the sample sizes were greater than 50–100. Furthermore, these uncertainties were close to those estimated using approximated normal distributions. Note that these results are based on the simulation study, and mathematical generalization remains unstudied. The resampling method for TDS curves can be used to expand the analyses of TDS tasks. In the future, resampling methods for a modified version of the TDS method, such as the TCATA method, need to be established, and successful analysis methods using the bootstrap resampling method are expected. For example, machine-learning methods using TDS curves to distinguish food products or judge food preferences are expected applications.

## Figures and Tables

**Table 1:** Tables of transition probabilities for Case 1 involving four descriptors. D*i* indicates descriptor *i*. Initial, middle, and last phases range $t^{\prime }=0$ – 6/20 , $t^{\prime }=7/20$ – 13/20 , and $t^{\prime }=14/20$ –1, respectively.

Initial	Destination				
Phase		D1	D2	D3	D4
From	D1	0.5	0.3	0.2	0
	D2	0.1	0.5	0.3	0.1
	D3	0.1	0.2	0.5	0.2
	D4	0	0	0.4	0.6
**Middle**	**Destination**				
**Phase**		**D1**	**D2**	**D3**	**D4**
From	D1	0.4	0.3	0.2	0.1
	D2	0.1	0.4	0.3	0.2
	D3	0.1	0.1	0.6	0.2
	D4	0.1	0.1	0.2	0.6
**Last**	**Destination**				
**Phase**		**D1**	**D2**	**D3**	**D4**
From	D1	0.2	0.2	0.3	0.3
	D2	0.1	0.3	0.3	0.3
	D3	0.1	0.2	0.3	0.4
	D4	0.1	0	0.2	0.7

**Table 2:** Transition tables for Case 2 involving six descriptors.

Initial	Destination						
Phase		D1	D2	D3	D4	D5	D6
From	D1	0.4	0.3	0.2	0.1	0	0
	D2	0.1	0.4	0.2	0.2	0.1	0
	D3	0.1	0.2	0.4	0.2	0	0.1
	D4	0	0.1	0.4	0.3	0.1	0.1
	D5	0	0	0.3	0.2	0.3	0.2
	D6	0	0	0.2	0.2	0.3	0.3
**Middle**	**Destination**						
**Phase**		**D1**	**D2**	**D3**	**D4**	**D5**	**D6**
From	D1	0.3	0.3	0.2	0.1	0.1	0
	D2	0.0	0.3	0.3	0.2	0.1	0.1
	D3	0.1	0.1	0.4	0.3	0.1	0
	D4	0	0.1	0.2	0.4	0.2	0.1
	D5	0	0	0.1	0.2	0.3	0.4
	D6	0	0	0.1	0.2	0.3	0.4
**Last**	**Destination**						
**Phase**		**D1**	**D2**	**D3**	**D4**	**D5**	**D6**
From	D1	0.1	0.2	0.3	0.3	0.1	0
	D2	0.1	0.3	0.3	0.3	0	0
	D3	0.1	0.2	0.3	0.4	0.1	0
	D4	0.1	0	0.2	0.5	0.2	0
	D5	0	0	0	0.1	0.6	0.3
	D6	0	0	0.1	0.1	0.3	0.5

## Abbreviations

The following abbreviations are used in this manuscript:

TCATA	temporal check-all-that-apply
TDS	temporal dominance of sensations
